# Intramural, Extra-endometrial Epithelial Tumor of the Uterine Corpus Associated With Adenomyosis: A Case Report of Clear Cell Carcinoma

**DOI:** 10.7759/cureus.79149

**Published:** 2025-02-17

**Authors:** Keiko Tabuchi, Shuichi Kurihara, Atsushi Takasugi, Yosuke Mizuno, Yumi Oshiro

**Affiliations:** 1 Department of Obstetrics and Gynecology, Saiseikai Fukuoka General Hospital, Fukuoka, JPN; 2 Department of Obstetrics and Gynecology, Japanese Red Cross Matsuyama Hospital, Matsuyama, JPN; 3 Department of Pathology, Japanese Red Cross Matsuyama Hospital, Matsuyama, JPN

**Keywords:** adenomyotic cyst, clear cell carcinoma, uterine adenomyosis, uterine cancer, who tumor classification

## Abstract

An increasing number of case reports have been published regarding the occurrence of extra-endometrial epithelial tumors of the uterine corpus. The majority of these cases are endometrial cancers arising in adenomyosis (EC-AIA). This report describes the case of a postmenopausal woman who presented with a cystic tumor measuring 15 cm in diameter in the pelvic cavity. Prior to surgery, the tumor was considered to be a malignant ovarian cystic tumor with solid components based on imaging studies. A laparotomy was performed, revealing an intramural tumor of the uterine corpus. The intraoperative frozen section diagnosis was inconclusive. Consequently, a hysterectomy with bilateral salpingo-oophorectomy was performed without additional staging procedures. The final histopathological examination by permanent section revealed that the tumor was histologically composed of clear cell carcinoma, accompanied by adenomyotic foci in the surrounding myometrium. It is noteworthy that while most uterine intramural tumors are mesenchymal in nature, intramural extra-endometrial epithelial tumors such as EC-AIA can also manifest as uterine intramural tumors.

## Introduction

A growing body of case reports has documented cases of epithelial tumors located within the wall of the uterine corpus, with intact endometrium free of tumor cells [1,2]. These tumors are rare, likely because the uterine myometrium normally lacks epithelial components. A review of the literature indicates that these intramural, extra-endometrial epithelial tumors of the uterine corpus, referred to as “IMEET-UC” in this report for convenience, include at least two distinct categories that show different histopathology and tumorigenesis. One of the two entities comprising IMEET-UC is a group of tumors that is generally referred to as “endometrial cancer arising in adenomyosis (EC-AIA)” in the literature [1]. As this terminology suggests, adenomyotic lesions within the uterine myometrium are believed to be the origin of this rare epithelial tumor. The other category that can manifest as IMEET-UC is mesonephric adenocarcinoma, which is even less prevalent than EC-AIA [2].

Because IMEET-UC typically does not involve the endometrium, preoperative endometrial sampling usually does not detect tumor cells [1,2]. Therefore, preoperative diagnosis and clinical decision-making mostly depend on imaging studies. Intraoperative frozen section histology may also be useful in determining how extensive or radical the surgical procedure should be. However, as this case report of EC-AIA demonstrates, interpretation of these two diagnostic modalities can be challenging, probably due to a lack of widespread awareness of these rare diseases.

## Case presentation

**Figure 1:**
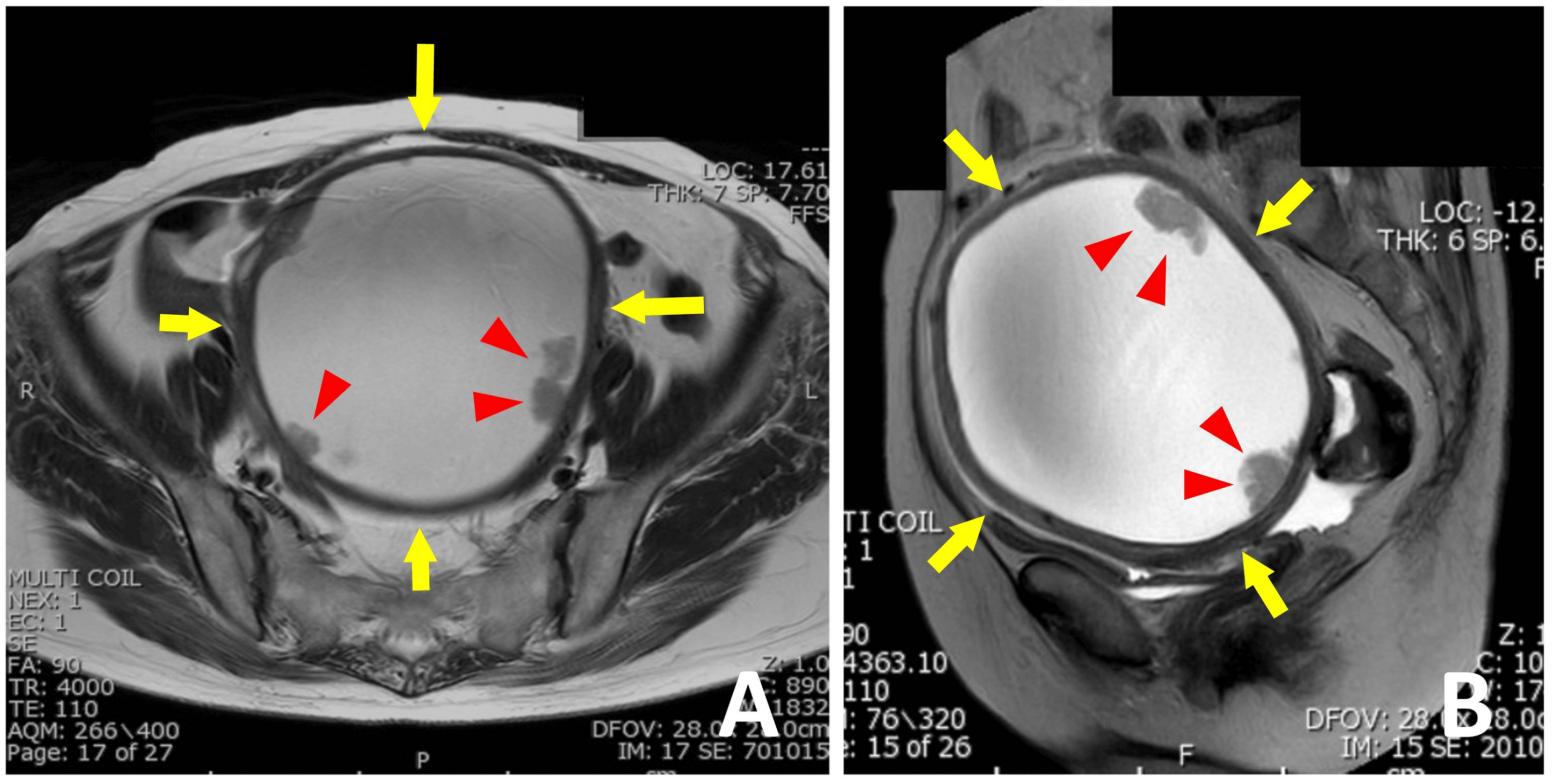
Magnetic resonance imaging of the pelvic cavity. Axial (A) and sagittal (B) T2-weighted MR images show a cystic tumor (arrows) with solid papillary components (arrowheads) protruding into the cyst lumen.

A 64-year-old Japanese postmenopausal woman, gravida 1, para 1, was referred to our hospital for evaluation of a pelvic mass, which was causing mild abdominal discomfort. Although the patient had a history of smoking (10 cigarettes/day for 20 years), she had no significant medical history. Her family history was unremarkable. There was no evidence of genital bleeding. A pelvic examination showed a well-mobile mass with a smooth surface. Vaginal ultrasound, abdominal CT, and pelvic MRI revealed a pelvic cystic tumor measuring 15 cm in diameter with multiple solid components protruding into the cyst lumen (Figure 1). The uterus, with unremarkable endometrium, was adjacent to the tumor, while the ovaries were not detectable in these imaging studies. The origin of the cystic tumor was indeterminate; both the uterus and ovaries were considered possible sites. A whole-body CT scan with contrast detected no metastatic lesions. With regard to serum tumor markers, the values of carbohydrate antigen 125 (CA-125), carbohydrate antigen 19-9 (CA 19-9), and carcinoembryonic antigen (CEA) were all within the normal range.

A presumptive diagnosis of an ovarian malignant tumor was made, and the patient underwent a laparotomy. A small amount of ascitic fluid was observed. The posterior wall of the uterine corpus, which had a smooth and intact surface, was enlarged by the tumor. The bilateral uterine adnexa appeared normal, and there was no evidence of peritoneal metastasis. The intraoperative frozen section of the uterine tumor revealed a lesion composed of tumor cells with clear cytoplasm, but a definitive histological diagnosis was not possible. Consequently, we performed abdominal hysterectomy and bilateral adnexectomy without staging procedures such as lymphadenectomy. Intraoperative cytology of the ascites was negative.

**Figure 2:**
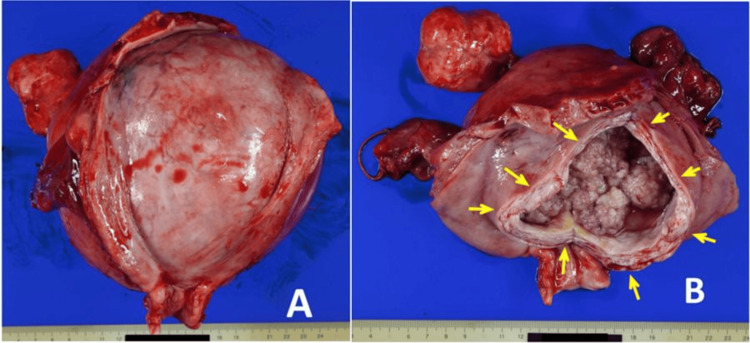
Gross appearance of the resected uterus. (A) The uterine anterior wall is cut and the uterine cavity is opened. The enlarged posterior wall of the uterine corpus contains a cystic tumor, which does not involve the endometrium. (B) The cyst wall is incised from the endometrial cavity and the cyst lumen is opened. This monolocular cystic tumor has multiple polypoid nodules protruding into the lumen. The arrows indicate the incision line.

Grossly, a 15 cm cystic lesion was present within the posterior wall of the uterine body, featuring cauliflower-like mural nodules protruding into the lumen (Figure 2).

**Figure 3:**
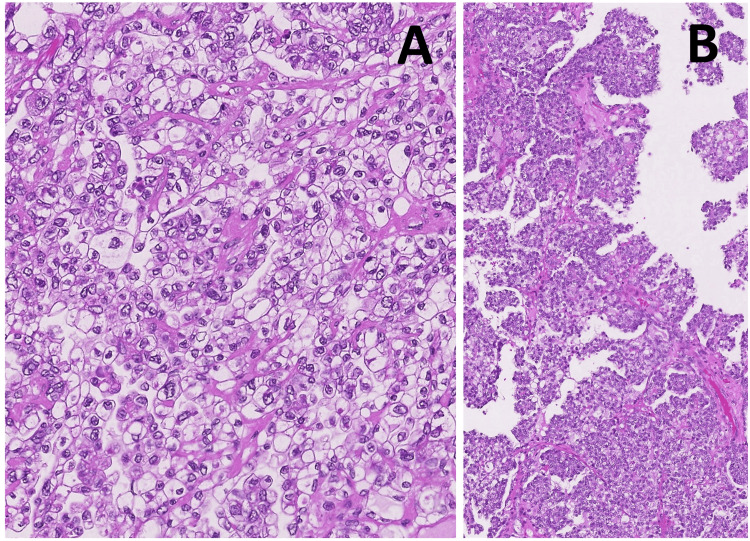
Microscopic findings of the tumor. (A) This specimen from the solid components protruding into the cyst lumen shows carcinoma cells with clear cytoplasm proliferating in solid sheets, (hematoxylin and eosin staining, original magnification x200). (B) In this area, the tumor cells are forming papillae (hematoxylin and eosin staining, original magnification x100).

The endometrium and the uterine serosa were smooth. Microscopically, the solid areas consisted of carcinoma cells with clear cytoplasm proliferating with papillary, tubular, and solid structures (Figure 3). These histological features of the uterine tumor were identical to those of ovarian clear cell carcinoma. The carcinoma cells invaded the surrounding myometrium, accompanied by lymphovascular invasion, while the endometrium remained intact.

**Figure 4:**
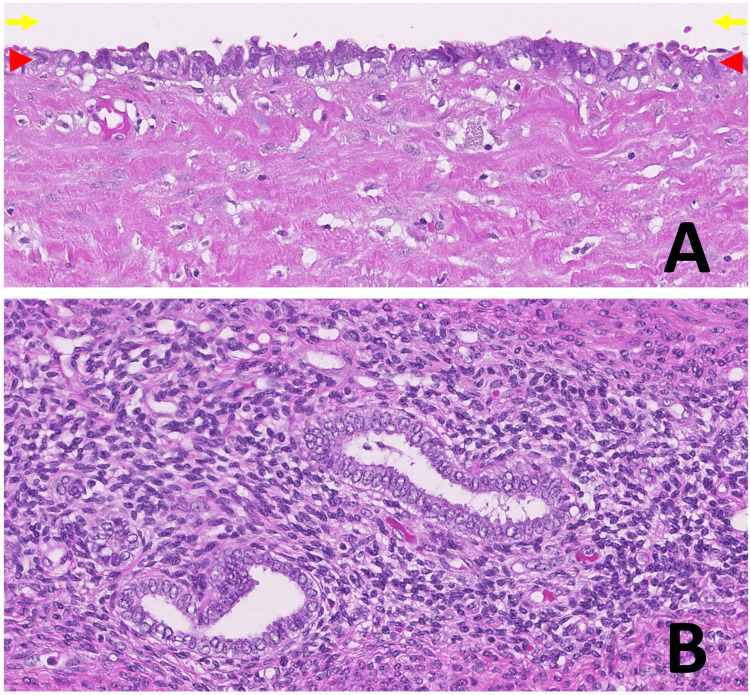
Microscopic findings of the cyst and the surrounding myometrium. (A) The inner surface of the cyst is lined by atypical epithelial cells. The arrows indicate the cyst lumen and the arrowheads show the epithelial layer, (hematoxylin and eosin staining, original magnification x200). (B) The surrounding myometrium contains adenomyotic foci consisting of endometrial glands and stroma, (hematoxylin and eosin staining, original magnification x200).

The inner surface of the cyst was primarily lined by a single layer of atypical epithelial cells, with areas lined by stratified epithelium (Figure 4A). These epithelial cells were surrounded by fibrous tissue with hyalinization. The uterine myometrium contained adenomyotic foci composed of endometrial glands without atypia and endometrial stroma (Figure 4B). The presence of cystic adenomyosis was not evident. No mesonephric remnants were identified in the myometrium or uterine cervix. The final pathological diagnosis was clear cell carcinoma originating in the wall of the uterine body. 

A postoperative CT scan revealed no discernible metastatic lesions. Following a comprehensive explanation of the risks and benefits associated with undergoing relaparotomy involving staging procedures, the patient indicated a preference for an alternative option that would exclude this additional surgery. Adjuvant chemotherapy with carboplatin and paclitaxel was administered, but multiple metastases occurred in the paraaortic and pelvic lymph nodes and the lungs three months after the surgery. Subsequently, she received another line of chemotherapy consisting of cisplatin and doxorubicin, but additional lymph node metastasis developed in the left subclavian region after she underwent the fourth cycle. Afterward, she was treated with lenvatinib and pembrolizumab. Later, multiple liver metastases emerged, and she died of the disease 25 months after the surgery.

## Discussion

In the present case, the lesion was confined to the myometrium of the uterine body without involvement of the endometrium. To the best of our knowledge, only two discrete pathological categories have been documented to present as IMEET-UC, like the current case, with the exception of metastatic tumors from other organs. The two groups comprising IMEET-UC are mesonephric adenocarcinoma of the uterine corpus [2-5] and endometrial cancer arising in adenomyosis (EC-AIA) [1,6-16].

Mesonephric adenocarcinoma in the female genital tract typically arises in the uterine cervix, and mesonephric remnants are believed to be the precursor of these tumors [17]. There have been documented cases of mesonephric adenocarcinoma occurring in the uterine body and presenting as IMEET-UC [2-5]. Consequently, this rare tumor should be considered in the histopathological differential diagnosis of IMEET-UC. It should be noted that some instances described as mesonephric adenocarcinoma of the uterine corpus in the literature may actually represent mesonephric-like carcinoma, a newly recognized entity in the most recent WHO Classification [17]. In the current case, histological findings suggestive of mesonephric adenocarcinoma or mesonephric-like adenocarcinoma, such as neoplastic tubules lined by cuboidal cells with lumina filled with dense eosinophilic secretions, were not detected. 

**Figure 5:**
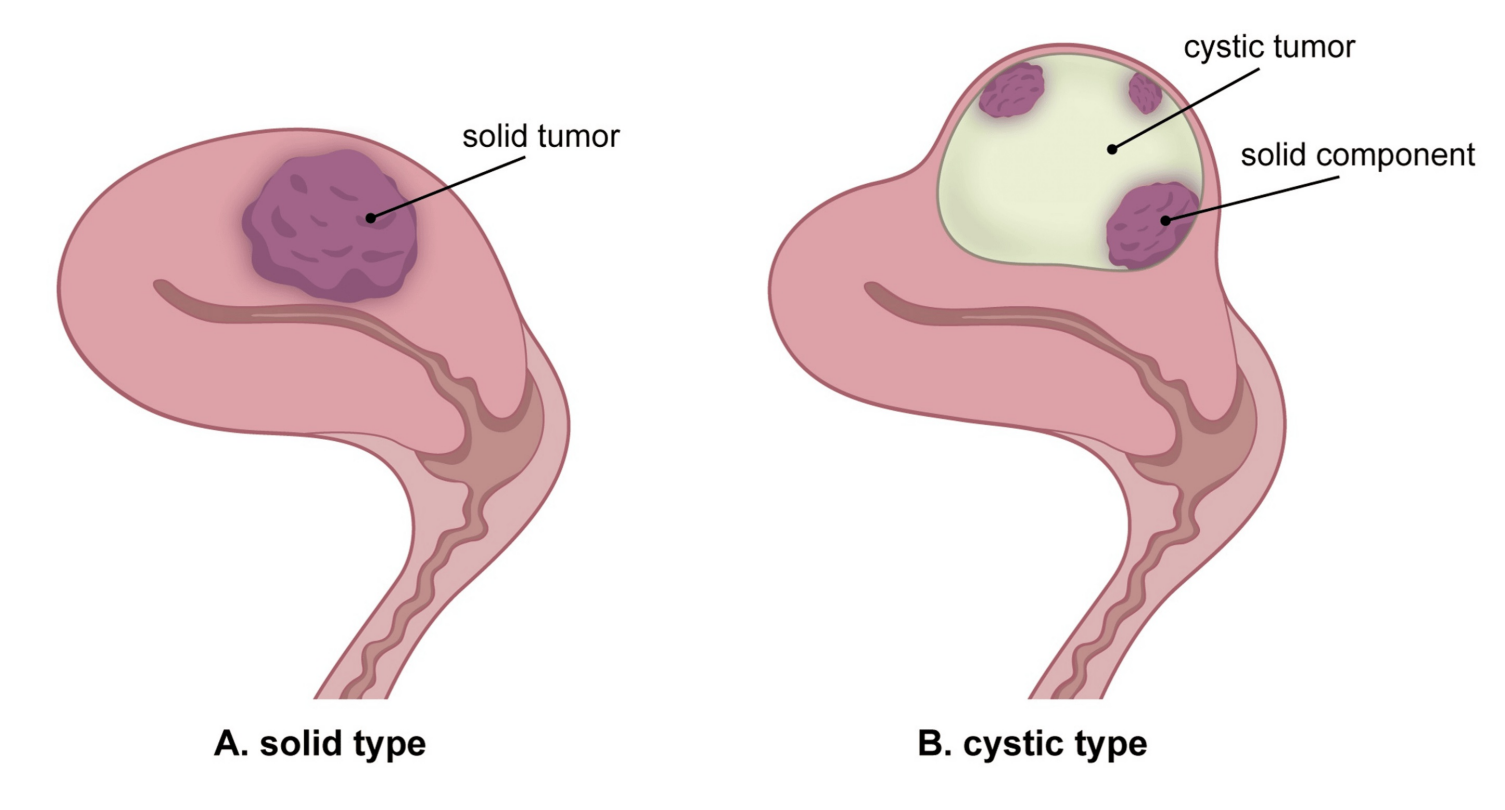
Schema of endometrial carcinoma arising in adenomyosis (EC-AIA) with representative morphologic features of the two subtypes. EC-AIA typically forms an intramural, extra-endometrial tumor in the uterine corpus. (The illustration is the original work of the authors.)

EC-AIA, another category presenting itself as IMEET-UC, has been considered to originate from adenomyosis [1]. Recent case reports and reviews have discussed this rare disease, and its clinicopathological features have become gradually understood. Izumi et al., a group of radiologists, reviewed the literature on MRI findings and proposed a practical subclassification of EC-AIA [13]. Izumi’s classification is helpful not only for the interpretation of MRI findings but also for understanding the essence of EC-AIA. To elucidate the representative clinicopathological characteristics of EC-AIA, it may be beneficial to simplify Izumi's three subtypes into two: the solid and the cystic types, as illustrated in Figure 5. Solid-type EC-AIA is macroscopically characterized by forming a solid tumor in the uterine wall [7,8,13]. The solid type has been reported more frequently than the cystic type.

On the other hand, to the best of our knowledge, there have only been nine case reports of cystic-type EC-AIA in the literature written in English [6,9-12,14-16,18]. These nine cases are summarized in Table 1 with clinicopathological features. As Izumi described, cystic-type EC-AIA typically forms a cystic mass with solid papillary projections within it [13]. Seven out of nine cystic-type EC-AIA cases in the literature, as well as our patient, demonstrated this macroscopic feature (Table 1). Retrospectively, the current case is considered a typical example of Izumi’s cystic-type EC-AIA in terms of MRI findings, gross appearance, and histological subtype.

**Table 1:** Clinicopathological features of cystic-type EC-AIA reported in the literature. BSO: bilateral salpingo-oophorectomy, LN: dissection of pelvic lymph nodes and/or para-aortic lymph nodes, mo: months.

References	Age	Preoprerative diagnosis	Surgery	Gross findings	Histological subtype	Normal or non-neoplastic epithelium lining the inner surface of the cyst wall, which suggests the presence of cystic adenomyosis	Presence of diffuse adenomyosis	Extrauterine spread	Clinical events/ outcomes (time interval between surgery and events, unless otherwise specified)	Sites of recurrence
Ohta et al. (2008) [9]	54	Ovarian cancer	Hysterectomy+ BSO+ Omentectomy	A cystic mass with solid areas	Clear cell carcinoma	Present ("cells with flattened nuclei showing minimal atypia")	(+)	Uterine and ovarian surface, omentum	Relapse (7 mo)	Liver
Baba et al. (2016) [12]	40	Uterine tumor with cystic degeneration or uterine malignant tumor	Hysterectomy	A cystic mass with solid areas	Clear cell carcinoma	Present ("normal endometrium")	(+)	(-)	No relapse (6 mo)	(-)
Gomez et al. (2021) [14]	65	Ovarian tumor	Hysterectomy+ BSO	A cystic mass with solid areas	Clear cell carcinoma	Not described	(+)	(-)	No relapse (13 mo)	(-)
Hoshiba et al. (2024) [15]	73	Malignant transformation of cystic adenomyosis	Hysterectomy+ BSO	A cystic mass with small, raised lesions on its inner surface	Clear cell carcinoma	Present ("columnar epithelium")	(+)	(-)	No relapse (6mo)	(-)
Morishita et al. (2024) [16]	51	Uterine leiomyoma with cystic degeneration	Myomectomy, followed by staging relaparotomy (hysterectomy+ BSO+ omentectomy+ appendectomy+ LN)	A cyst within a leiomyoma	Clear cell carcinoma	Not described	Not described	Para-aortic lymph nodes	Relapse (22 mo after completing chemotherapy), Death (13 mo after the diagnosis of recurrence)	Liver, supraclavicular lymph nodes
Ichikawa et al. (2001) [6]	35	Ovarian cancer	Hysterectomy+ BSO+ LN	A cystic mass with solid areas	Endometrioid carcinoma	Not described	(+)	(-)	No relapse (42 mo)	(-)
Heo et al. (2011) [10]	54	Uterine tumor with cystic degeneration or uterine malignant tumor	Hysterectomy+ BSO	A cystic mass with solid areas	Endometrioid carcinoma	Present ("adenomyotic glandular epithelium")	Not described	(-)	No relapse (12 mo)	(-)
Mori et al. (2015) [11]	67	Uterine tumor with cystic degeneration or uterine malignant tumor	Hysterectomy+ BSO	A cystic mass with solid areas	Endometrioid carcinoma	Absent	(+)	(-)	No relapse (16 mo)	(-)
Kawamura et al. (2014) [18]	38	Ovarian tumor	Hysterectomy+ BSO	A cystic mass with solid areas	Seromucinous borderline tumor (Mullerian-type mucinous borderline tumor)	Present ("a single layer of either mucinous or endometrial epithelium without atypia")	(+)	(-)	No relapse (24 mo)	(-)
Current case	64	Ovarian tumor	Hysterectomy+ BSO	A cystic mass with solid areas	Clear cell carcinoma	Absent	(+)	(-)	Relapse (3 mo), Death (25 mo)	Lung, pelvic, and paraaortic lymph nodes

From an etiological perspective, Hoshiba et al. recently proposed a hypothesis describing two distinct mechanisms underlying the malignant transformation of adenomyosis into EC-AIA [15]. The first group of neoplasms, presumed to originate from diffuse adenomyosis, corresponds to Izumi’s solid type. Endometrioid carcinoma is the most prevalent histological subtype in this solid-type EC-AIA. Hoshiba’s second group comprises lesions believed to arise in cystic adenomyosis, aligning with Izumi’s cystic type. Hoshiba postulated that these cystic EC-AIA become malignant through a mechanism similar to that of Mullerian-type ovarian epithelial tumors developing in endometriotic cysts. Not only the gross appearance but its histological features also resemble those of ovarian Mullerian epithelial tumors arising in an endometriotic cyst; the two main histological subtypes of cystic-type EC-AIA are clear cell carcinoma and endometrioid carcinoma (Table 1). Notably, a case of seromucinous borderline tumor, which was previously called Mullerian-type mucinous borderline tumor, has been documented in the literature [18], lending further support to Hoshiba’s hypothesis that cystic-type EC-AIA may develop through a similar etiology to that of Mullerian-type ovarian epithelial tumors arising in endometriotic cysts.

Out of nine reported cases, five showed normal or non-neoplastic epithelium lining the cyst wall, indicating the presence of cystic adenomyosis, while one case did not. In the present case, we could not detect the definitive component of benign cystic adenomyosis. There is a possibility that the pre-existing non-neoplastic tissue of an adenomyotic cyst was entirely or largely replaced by cancerous tissue in this case. Alternatively, there may be another way of forming a cystic-type EC-AIA other than the malignant transformation of an adenomyotic cyst. 

Clinically, it is noteworthy that preoperative diagnosis of IMEET-UC, such as EC-AIA, depends on imaging studies in most cases because endometrial sampling with histological or cytological examination does not detect tumor cells until the lesion invades the endometrium. Currently, MRI is the most important diagnostic tool for diagnosing these uterine disorders [13]. In the current case, we had preoperatively considered her intrapelvic cystic tumor as an ovarian cystic tumor based on MRI findings, highlighting the difficulty of MRI interpretation of EC-AIA. According to the review by Raffone et al., many cases of EC-AIA were preoperatively diagnosed as uterine mesenchymal tumors such as sarcoma and leiomyoma, or ovarian cancer [1]. Regarding cystic-type EC-AIA, four out of nine cases were preoperatively diagnosed as ovarian tumors, similar to the patient described in the current report (Table 1). More widespread awareness regarding EC-AIA is desirable; a recent review of MRI findings of malignant uterine myometrial tumors, written for educational purposes for radiologists, included a section about EC-AIA [19]. This suggests that EC-AIA is beginning to be noticed by radiologists as an important disease that can be a pitfall in MRI diagnosis of uterine myometrial tumors.

Due to the challenges inherent in preoperative diagnosis, intraoperative frozen section histology can play a crucial role in determining surgical procedures for cases suspected preoperatively of being IMEET-UC. For instance, clinicians may be able to decide whether to perform staging procedures such as lymphadenectomy for lesions that are grossly confined to the uterus. In the current case, intraoperative frozen section histology did not confirm the diagnosis, leading to surgery with incomplete surgical staging. A potential explanation for the ineffectiveness of intraoperative frozen section diagnosis in the present case is the limited awareness of this rare disease. Although IMEET-UC, such as EC-AIA, is not explicitly mentioned in the current WHO Classification of Tumors (fifth edition) [17], recognizing IMEET-UC, such as EC-AIA, as a distinct disease entity could be clinically beneficial.

The current case demonstrated chemoresistance, as evidenced by the recurrence of the tumor during the administration of initial adjuvant chemotherapy. Among the nine reported cases of cystic-type EC-AIA in the literature, two patients demonstrated extrauterine tumor spread (Table 1). These two individuals also experienced tumor relapse. On the other hand, two patients lived for more than two years without recurrence. Therefore, early detection and surgical intervention may be beneficial for cases of cystic-type EC-AIA. 

## Conclusions

It is noteworthy that while the majority of uterine intramural tumors are mesenchymal in nature, intramural extra-endometrial epithelial tumors such as EC-AIA can also manifest as uterine intramural tumors. Preoperative imaging studies, particularly MRI and intraoperative frozen section analysis, are crucial for choosing adequate management of these diseases. Clinicopathological characteristics of EC-AIA are gradually being understood, but broader recognition is still needed.
